# INFANTILE IATROGENIC CUSHING'S SYNDROME

**DOI:** 10.4103/0019-5154.44793

**Published:** 2008

**Authors:** Selahattin Katar, Sedat Akdeniz, M Nuri Özbek, Ahmet Yaramiş

**Affiliations:** *From the Department of Pediatrics, Dicle University Faculty of Medicine, Diyarbakir, Turkey*; 1 *From the Department of Dermatology, Dicle University Faculty of Medicine, Diyarbakir, Turkey*

**Keywords:** *İatrogenic Cushing's syndrome*, *topical corticosteroid*, *child*

## Abstract

High potency or/and extended use of topical corticosteroids, particularly in children, may cause suppression of the hypothalamopituitary-adrenal axis. However, iatrogenic Cushing's syndrome in infantile age group is very rare and only a few patients have been reported to date in the literature. Here, we report a case of iatrogenic Cushing's syndrome in a 6-month-old male child whose parents have admitted to the hospital for overweight and skin fragility.

## Introduction

Iatrogenic Cushing's syndrome may occur with the overuse of potent topical steroids.^1^,^2^ Corticosteroids are divided into four groups with respect to clinical potency according to the United States Pharmacopeia USP. Only those labeled as low potency are acceptable for chronic use in infants and young children. High-potency local steroids are used primarily as an alternative to the systemic corticosteroids, although local absorption of these products may lead to severe systemic effects.^3^

Children have greater risks of local and systemic side effects of topical steroids as they have higher body surface area. For this reason, these products should better not to be purchased from the drugstore without prescription. Also, information about the usage period and probable side effects of product should be given to the parents.

A case of a 6-month-old child with iatrogenic Cushing's syndrome due to prolonged application of very high-potency topical corticosteroid pomade clobetasol 17-propionate is presented.

## Case Report

A 6-month-old male child was admitted to our hospital with a 3-month history of overweight. His family history revealed that he is the third living child. He had two healthy brothers, aged four and seven. His brothers and other members of the family had no similar disease.

**Figure 1:**
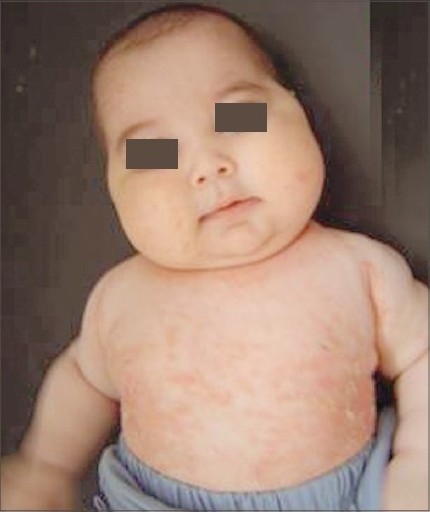
Cushingoid features of patient after 2 months of topical steroid application

On physical examination, axillary temperature was 37°C, blood pressure was 111/68 mmHg and heart rate was 98/min. His weight was 8600 g 75–90th percentile, height 65 cm 25–50th percentile, and head circumference 42 cm 10–25th percentile. He had Cushingoid image, truncal obesity, drumstick limbs, and paper-thin skin with striae, and crops of dense, inflamed pustules on his chest and back (Fig. 1).

On laboratory examination – hemoglobin: 14.6 g/dl, white blood cell: 16,600/mm^3^, platelet: 614,000/mm^3^, serum glucose: 91 mg/dl, blood urea: 25 mg/dl, creatinine: 0.4 mg/dl, sodium: 142 mEq/L, potassium: 4 mEq/L, chloride: 105 mEq/L.

Morning cortisol level was low, 0.66 μg/dl. Adrenocorticotropic hormone (ACTH) was not elevated, 7.1 pg/ml. The peak cortisol level after the standard dose of 250 μg/m^2^ ACTH was 18.2 μg/dl.

When parents were questioned for the details of patient's history, we learned about the usage of pomade clobetasol-17-propionate (3 tubes, each of 30g). It was used two to three times in a day for the last 3 months for skin dryness. This product was purchased from drugstore without any prescription. The parents were not informed about the application period and possible side effects of the product.

## Discussion

Topical corticosteroids have been ordered extensively in medical practice by many doctors for various skin diseases. If information about their side effects are not given to the parents, the extended use of high-potency topical corticosteroids in children may cause severe local and systemic side effects. Systemic and local side effects include hypertension, Cushing's syndrome, hypothalamopituitary-adrenal axis suppression, failure to thrive, glaucoma, cataract, skin atrophy, striae and predisposition to the bacterial, and fungal infections.^4^

Extensive and prolonged misuse of potent topical steroid preparations in children may cause suppression of ACTH, with increased need of endogen cortisol production.^5^,^6^ In presented case, Cushing's syndrome developed due to the improper use of topical corticosteroid. Paper-thin skin with striae, crops of dense, inflamed pustules on chest and back and hypertension was the positive physical signs of Cushing's syndrome of our patient. In addition to these signs, parents gave the history of recurent respiratory system infections.

The application of very high-potency topical corticosteroids, particularly in children, may cause immune suppression, and this suppression may lead a tendency to bacterial and fungal infections.^7^ Şiklar *et al.*^1^ reported a case of urinary infection due to inappropriate use of topical corticosteroid.

In pediatric patients, large amounts of topical corticosteroids may be absorbed from the skin in a short period as the ratio of body surface area to the weight is greater than adult. In our case, mild hypertension to the local steroid treatment was recorded and the blood pressure turned into normal limits after the cessation of application. Cushing syndrome development in children due to prolonged usage of topical corticosteroids have been reported in literature.^1^,^8^

In our case, morning cortisol level was 0.66 μg/dl and ACTH level was 7.1 pg/ml. The response to ACTH stimulation was not sufficient.

Highly potent topical products should not be used in infants, and information about possible side effects of topical corticosteroids should be given to the parents. The local and general health committees have to take consideration about the misuse of the potent and highly potent topical corticosteroids application. Precaution should be taken for the topical corticosteroids use, particularly in pediatric patients, its quantity, application time and prescription obligation.

The history of the topical corticosteroids should be reminded in children with Cushing's appearance.
